# The protocol-guided rapid evaluation of veterans experiencing new transient neurological symptoms (PREVENT) quality improvement program: rationale and methods

**DOI:** 10.1186/s12883-019-1517-x

**Published:** 2019-11-20

**Authors:** D. M. Bravata, L. J. Myers, B. Homoya, E. J. Miech, N. A. Rattray, A. J. Perkins, Y. Zhang, J. Ferguson, J. Myers, A. J. Cheatham, L. Murphy, B. Giacherio, M. Kumar, E. Cheng, D. A. Levine, J. J. Sico, M. J. Ward, T. M. Damush

**Affiliations:** 1 Department of Veterans Affairs (VA) Health Services Research and Development (HSR&D), Precision Monitoring to Transform Care (PRISM) Quality Enhancement Research Initiative (QUERI), Indianapolis, USA; 2 VA HSR&D Center for Health Information and Communication (CHIC), Richard L. Roudebush VA Medical Center, HSR&D Mail Code 11H, 1481 West 10th Street, Indianapolis, IN 46202 USA; 3 Department of Internal Medicine, Indiana University School of Medicine, Indianapolis, IN USA; 4 Department of Neurology, Indiana University School of Medicine, Indianapolis, IN USA; 5 Regenstrief Institute, Indianapolis, IN USA; 6 Department of Biostatistics, Indiana University School of Medicine, Indianapolis, IN USA; 7 Department of Biostatistics, University of Nebraska Medical Center, Omaha, NE USA; 8 Office of Healthcare Transformation (OHT), Veterans Health Administration (VHA), Washington, DC USA; 9 Department of Neurology, VA Greater Los Angeles Healthcare System, California, Los Angeles USA; 10 Department of Neurology, David Geffen School of Medicine, University of California at Los Angeles, California, Los Angeles USA; 11 Department of Internal Medicine and Neurology and Institute for Health Policy and Innovation, University of Michigan School of Medicine, Ann Arbor, MI USA; 12 Clinical Epidemiology Research Center and Neurology Service, VA Connecticut Healthcare System, West Haven, CT USA; 13 Departments of Internal Medicine and Neurology and Center for Neuroepidemiology and Clinical Neurological Research, Yale School of Medicine, New Haven, CT USA; 14 VA Tennessee Valley Healthcare System, Nashville, TN USA; 15 Department of Emergency Medicine, Vanderbilt University Medical Center, Nashville, TN USA

**Keywords:** Cerebrovascular disease, Transient ischemic attack, Learning healthcare system, Quality of care, Implementation science, Audit and feedback, Systems redesign

## Abstract

**Background:**

Transient ischemic attack (TIA) patients are at high risk of recurrent vascular events; timely management can reduce that risk by 70%. The Protocol-guided Rapid Evaluation of Veterans Experiencing New Transient Neurological Symptoms (PREVENT) developed, implemented, and evaluated a TIA quality improvement (QI) intervention aligned with Learning Healthcare System principles.

**Methods:**

This stepped-wedge trial developed, implemented and evaluated a provider-facing, multi-component intervention to improve TIA care at six facilities. The unit of analysis was the medical center. The intervention was developed based on benchmarking data, staff interviews, literature, and electronic quality measures and included: performance data, clinical protocols, professional education, electronic health record tools, and QI support. The effectiveness outcome was the without-fail rate: the proportion of patients who receive all processes of care for which they are eligible among seven processes. The implementation outcomes were the number of implementation activities completed and final team organization level. The intervention effects on the without-fail rate were analyzed using generalized mixed-effects models with multilevel hierarchical random effects. Mixed methods were used to assess implementation, user satisfaction, and sustainability.

**Discussion:**

PREVENT advanced three aspects of a Learning Healthcare System. Learning from Data: teams examined and interacted with their performance data to explore hypotheses, plan QI activities, and evaluate change over time. Learning from Each Other: Teams participated in monthly virtual collaborative calls. Sharing Best Practices: Teams shared tools and best practices. The approach used to design and implement PREVENT may be generalizable to other clinical conditions where time-sensitive care spans clinical settings and medical disciplines.

**Trial registration:**

clinicaltrials.gov: NCT02769338 [May 11, 2016].

## Background

With the proliferation of electronic health records and increased emphasis on Learning Healthcare Systems, healthcare teams are being tasked with responding to data-driven quality problems [1]. Teams may deploy a variety of quality improvement (QI) strategies and systems redesign approaches to improve performance, depending on the complexity and scope of the problem. This description of the rationale, implementation strategy, and evaluation plan of the Protocol-guided Rapid Evaluation of Veterans Experiencing New Transient Neurological Symptoms (PREVENT) trial details an approach to developing and evaluating a multi-component QI intervention for a complex, time-sensitive clinical problem that involves several clinical disciplines and is consistent with the principles of the Learning Healthcare System model. This report adheres to the Revised Standards for Quality Improvement Reporting Excellence (SQUIRE 2.0) [2, 3].

### The problem being addressed

Approximately 8500 Veterans with transient ischemic attack (TIA) or ischemic stroke are cared for in a Department of Veterans Affairs (VA) Emergency Department (ED) or inpatient ward annually in the United States [4]. Patients with TIA generally present with transient neurological symptoms of presumed ischemic etiology [5]. TIA patients at high risk of recurrent vascular events [6–8], however, interventions which deliver timely TIA care can reduce that risk by up to 70% [9–12]. Despite the known benefits of timely TIA care, data from both selected private-sector United States hospitals (i.e., facilities that have implemented stroke quality improvement programs) [13] and from the VA healthcare system have identified gaps in TIA care quality. For example, only 51% of Veterans who were eligible received carotid imaging as part of their TIA care [14]. Moreover, the majority of VA facilities do not have a TIA-specific protocol [15].

### Objective

The objective of the PREVENT trial was to develop, implement, and evaluate a multi-component, QI intervention to improve the quality of care for Veterans with TIA that could be scaled to serve the full spectrum of VA medical centers, ranging from small facilities with few specialist resources to the most complex and well-resourced facilities with access to comprehensive academic medical centers. The Consolidated Framework for Implementation Research (CFIR) guided the development of the PREVENT intervention, its accompanying implementation strategies, and its evaluation plan [16, 17]. Our approach contributed to the development of a Learning Healthcare System and may be generalizable to QI interventions that target healthcare teams [18].

## Methods

### Context

Within the VA, quality measurement and systems redesign are integrated into the healthcare system within administration and clinical operations [19, 20]. Although stroke care quality metrics are reported, there is currently no VA system-wide focus on TIA care quality. TIA is a clinical condition that is relatively common and for which there is a time-sensitive imperative to provide diagnostic and management processes of care. However, there is no existing VA quality measurement or “top-down” mandate for QI related to TIA care. Nevertheless, because of the demonstrable gaps in the quality of TIA care for Veterans, VA leadership, namely in neurology and emergency medicine, provided robust support for a TIA quality improvement program.

### Quality improvement intervention development

The development of the PREVENT intervention [21–24] was based on a systematic assessment of TIA care performance at VA facilities nationwide as well as critical barriers and facilitators of TIA care performance using four sources of information: baseline quality of care data [14], staff interviews [15], existing literature [25–28], and validated electronic quality measures [14].

#### Baseline quality of care data

The first national benchmarking study of TIA care quality in the VA included patients cared for in any VA ED or an inpatient setting during federal-fiscal year 2014 [29]. Among *N* = 8201 patients in 129 facilities, performance varied across elements of care from brain imaging within 2 days of presentation (88.9%) to high/moderate potency statin within 7 days post-discharge (47.2%). Performance also varied substantially across facilities. Performance was higher for admitted patients than for patients cared for only in EDs, with the greatest disparity for carotid artery imaging: 75.6% versus 25.3% (*p* < 0.0001). These data provided justification for developing a QI project to improve TIA care quality.

#### Staff interviews

**Fig. 1:**
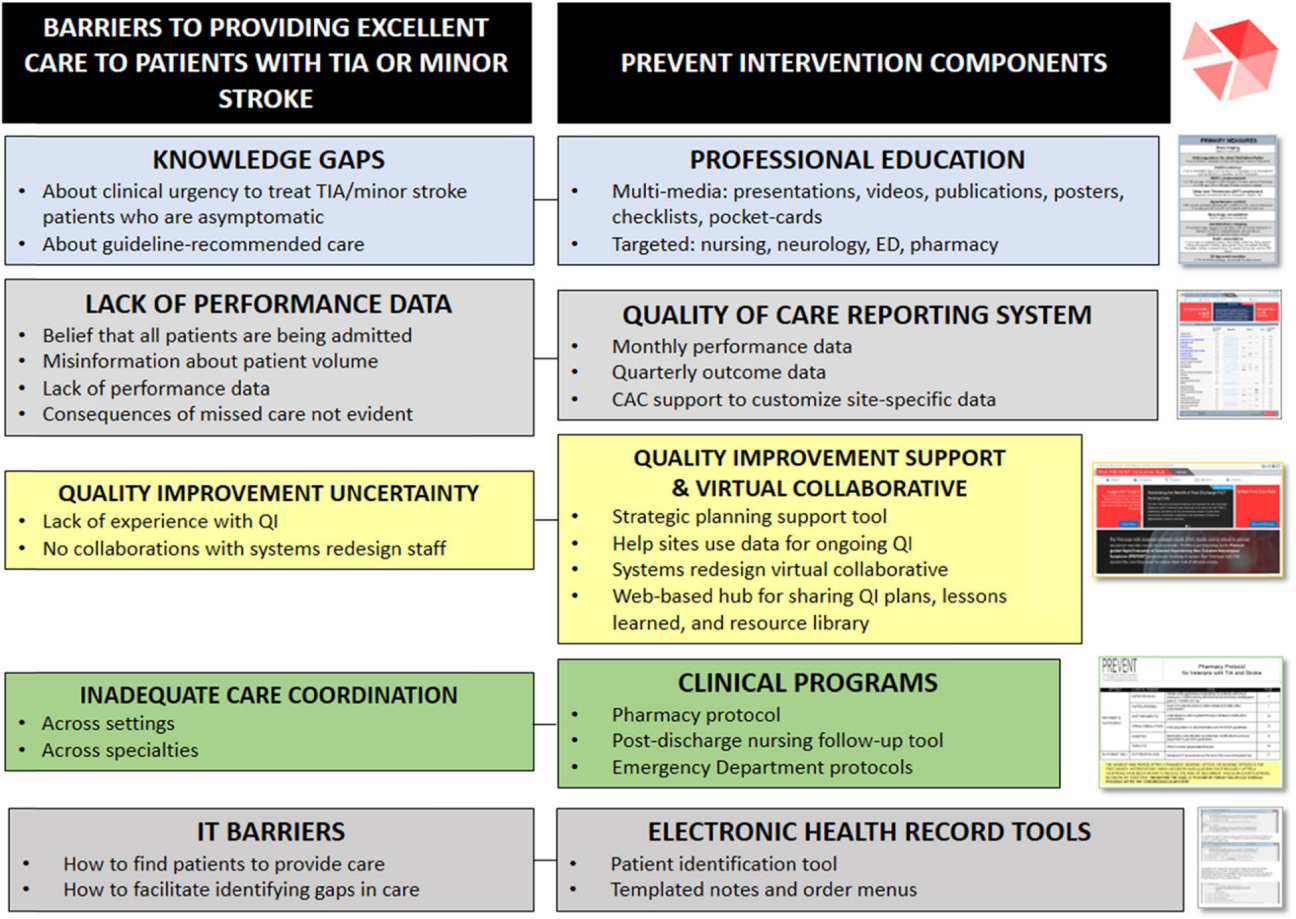
Intervention Components Mapped onto Barriers to Providing Quality Care. Figure 1 displays the barriers to providing excellent quality of care for patients with transient ischemic attack (TIA) or minor stroke that were identified through interviews with front-line clinicians as well as the components of the PREVENT program that were designed to overcome each barrier

Interviews with staff members involved in the care of patients with TIA from multiple disciplines (neurology, emergency medicine, nursing, pharmacy, primary care, hospitalist medicine, radiology, vascular surgery, cardiology, ophthalmology, systems redesign, and quality management) at 14 diverse VA facilities identified barriers to providing high quality TIA care including: gaps in knowledge, lack of performance data, uncertainty about how to engage in QI, inadequate care coordination, and information technology barriers [15, 29]. The PREVENT intervention was designed to address these barriers by drawing upon existing VA resources (e.g., staffing, VA data systems, etc) (Fig. 1).

#### Prior literature

Several studies have demonstrated that providing timely diagnosis and management improves care and outcomes for patients with TIA [9–12, 27, 28]. For example, three effectiveness studies included algorithms or protocols that facilitated the timely delivery of care for patients with TIA. Based on this research, PREVENT included algorithms and protocols to promote timely delivery of the guideline-concordant processes of care that have been associated with improved outcomes [9].

#### Validated electronic quality measures

Electronic quality measures were developed using electronic health record data and were validated against chart review [30]. A random sample of 763 TIA or minor ischemic stroke patients cared for in 45 VA facilities was used to construct electronic versions of 31 existing quality measures [30]. The measures with the most robust performance against chart review became the PREVENT measures [30].

### Quality improvement intervention description

The PREVENT QI intervention targeted facility providers not individual patients. External facilitation was provided by the study team, which included a nurse (with quality management and clinical nursing experience), a general internist (with QI and stroke clinical care experience), implementation scientists (from diverse backgrounds including health psychology, education and medical anthropology), and a senior data scientist. The participating facility teams were diverse but generally included members from neurology, emergency medicine, nursing, pharmacy, and radiology; some teams also included hospitalists, primary care staff, education staff, telehealth staff, ophthalmologists, or systems redesign staff. The primary site champion was the person designated as being responsible for stroke care quality at the participating facility. Therefore, for the majority of sites, the champion was a neurologist, but at one site the champion was an ED nurse and at another site the role of champion was shared by staff from neurology and pharmacy. The PREVENT QI intervention included five components [1]: quality of care reporting system [2], clinical programs [3], professional education [4], electronic health record tools, and [5] QI support including a virtual collaborative (Fig. 1).

#### Quality of care reporting system: audit and feedback

**Fig. 2:**
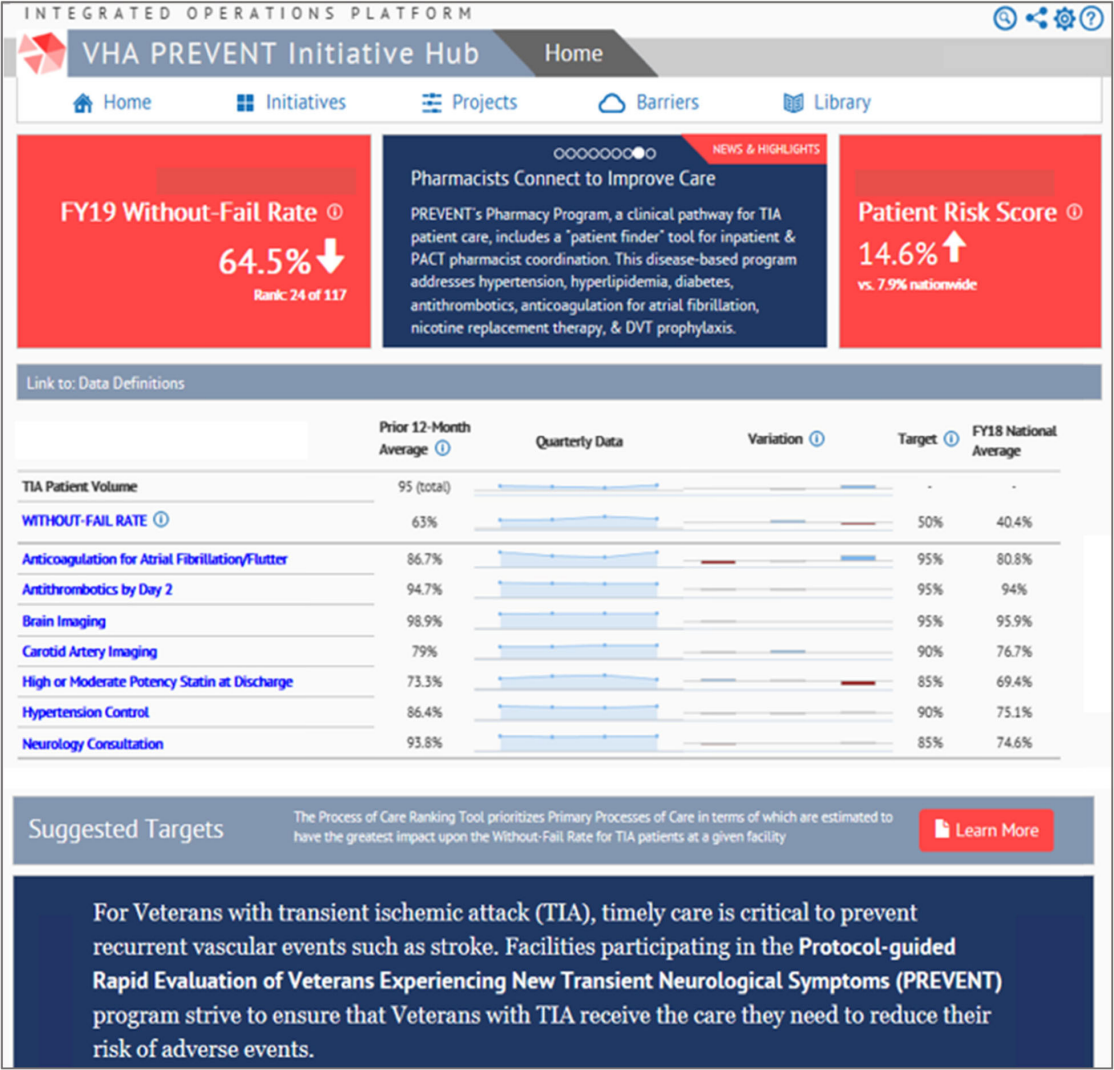
The PREVENT Web-Based Hub. The home page of the web-based PREVENT Hub included a prominent display of the facility without-fail rate (upper left red box) and the pass rates for each of the seven key processes of care (in blue text)

The web-based PREVENT Hub (Fig. 2) provided data about a broad range of processes of care (e.g., brain imaging), healthcare utilization (e.g., proportion of patients with a primary care visit within 30-days of the index TIA), and other aspects of care (e.g., proportion of TIA patients who left against medical advice). These data were updated monthly for every VA facility. Aggregated data were presented at the facility level (not the patient or provider level) and placed in context by being displayed alongside suggested targets and VA national rates. The PREVENT Hub allowed users to customize views to examine quality over time and to compare themselves with other facilities. Users could explore hypotheses about whether their performance varied for patients who presented on weekdays versus weekends, for patients who were admitted to the hospital versus discharged from the ED, or for patients with neurology consultation versus without neurology consultation.

#### Clinical protocols

Several clinical programs were developed and shared on the PREVENT Hub. For example, a pharmacist-based TIA medication management protocol was developed to improve medication-related processes of TIA care (e.g., hypertension and hyperlipidemia management). The pharmacy protocol utilized existing VA pharmacy staff in the inpatient or ED settings with hand-offs to pharmacists embedded in the primary care teams. In addition, a templated note and checklist were created for VA primary care nurses. PREVENT site teams developed ED-based protocols for TIA patients which were also shared on the PREVENT Hub.

#### Professional education

The PREVENT staff education materials were diverse, including: slide sets (with speaker notes) designed specifically for physicians and residents, pharmacists, and nurses; guidelines and article reprints; videos (one described the importance of providing timely TIA care and one demonstrated a clinical team reflecting on quality of care data, evaluating progress toward goals, and planning QI activities in response to data); as well as pocket-cards and posters. Locally-generated educational materials were also shared on the Hub.

#### Electronic health record tools

A variety of electronic health record tools were available for PREVENT sites to adapt including: order menus, note templates, and a patient identification tool. The note templates were developed using reminder dialogues to enable teams to monitor when templates were used. The patient identification tool was developed to identify individual TIA patients who were seen in a facility in the ED or inpatient ward so that the site teams could ensure that highest quality care was being delivered in real time (as opposed to waiting for retrospective data).

#### Quality improvement support & virtual collaborative

Active implementation of PREVENT involved a full-day kickoff meeting. The kickoff included all relevant staff members at a participating site and study team members, some participated in person and others participated via videoconference. The kickoff was designed to be fun, engaging, educational, and productive. The PREVENT study team members explicitly developed the agenda with the belief that the most important resource for the kickoff was the time and attention of the participating staff members, with the event providing a crucial opportunity for team formation (at many sites team members were meeting each other for the first time at the kickoff).

The kickoff began with presentations, videos, and activities to create a sense of excitement and empowerment about improving care and outcomes for patients with TIA. The facility team used the PREVENT Hub to explore their facility-specific quality of care data and identify processes of care with the largest gaps in quality for the greatest number of patients. Using approaches from systems redesign, facility team members brainstormed about barriers to providing highest quality of care, identified solutions to address barriers, ranked solutions on an impact-effort matrix, and developed a site-specific action plan that included high-impact/low-effort activities in the short-term plan and high-impact/high-effort activities in the long-term plan. Throughout the kickoff, the facility team was introduced to PREVENT components (e.g., videos from the education program and the pharmacy clinical protocol) as well as strategies for engaging in key QI activities such as reflecting and evaluating, goal setting, and planning.

Local QI plans were entered into the PREVENT Hub, and metrics were tracked allowing teams to monitor performance over time. PREVENT site teams could learn from the overall community by identifying which QI activities either did or did not achieve improvement in metrics at other sites.

During the one-year active implementation period, the teams joined monthly PREVENT collaborative conferences which served as a forum for facility team members to share progress on action plans, articulate goals for the next month, and review any new evidence or tools [31]. The monthly collaborative conferences were conducted via a shared meeting platform that allowed for screen sharing and instant messaging; videoconferencing was also occasionally used. During each collaborative conference, invited speakers with expertise related to cerebrovascular risk factor management, VA healthcare administration, or systems redesign reviewed topics of interest using cases to stimulate discussion, identify barriers, and brainstorm about solutions. Participants received continuing education credits. At the end of the one-year active implementation period, the collaborative call was conducted via video-conference and was used to acknowledge the implementation accomplishments of the site which was being promoted from active implementation to sustainability. Facility leadership was invited to celebrate the successes of the local team.

### Evaluation approach

**Fig. 3:**
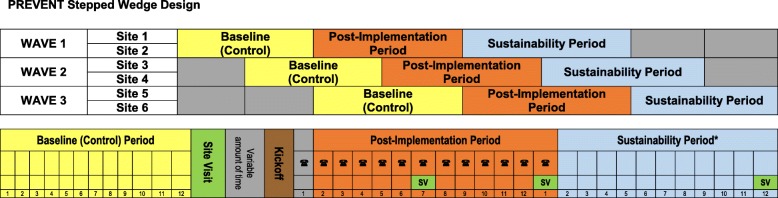
PREVENT Stepped-wedge Design. The stepped-wedge design included two sites per wave and a total of three waves. The study included: a 12-month baseline period (yellow); a12-month post-implementation period (orange), which began with a kick-off (brown), and during which the site teams participated in monthly virtual collaborative sessions (telephone symbol); and a sustainability period (blue)

A five-year stepped-wedge [32, 33] Hybrid Type II [34] implementation trial included six participating sites where active implementation was initiated in three waves, with two facilities per wave (Fig. 3). The unit of analysis was the VA facility. Stepped-wedge designs are increasingly being used in health services and implementation research when the intervention is not implemented at the individual patient level but is rather implemented sequentially within participating clusters [21, 22, 35, 36]. In stepped-wedge designs, all of the clusters (i.e., individual participating VA facilities) begin with a control (baseline) condition and then initiate the intervention as the study progresses. The PREVENT trial involved three phases: a 1 yr-baseline period, a one-year active implementation period (that began 13 months after the start of the baseline period, providing 1 month for facility teams to initiate QI activities), and a 1-year sustainability period (following the end of active implementation; Fig. 3). The evaluation involved four assessments of PREVENT: effectiveness, users’ assessment, implementation, and sustainability. PREVENT was registered with clinicaltrials.gov (NCT02769338) and received human subjects (institutional review board [IRB]) and VA research and development committee approvals.

#### Primary effectiveness outcome

The primary effectiveness outcome was the “without-fail” rate, defined as the proportion of Veterans with TIA who received all of the processes of care for which they were eligible from among seven processes of care: brain imaging, carotid artery imaging, neurology consultation, hypertension control, anticoagulation for atrial fibrillation, antithrombotics, and high/moderate potency statins [37]. These seven measures were included in the without-fail rate because they are both guideline-recommended processes of care and they have been associated with improvements in TIA patient outcomes [37]. The without-fail rate is sometimes also referred to as “defect-free” care [38, 39]. It is an all-or-none measure of quality, which assesses for an individual patient whether they either did (“passes” the without-fail measure), or did not (“fails” the without-fail measure) receive all of the elements of care for which they were eligible. The without-fail rate was calculated at the facility level based on electronic health record data using validated algorithms [14].

The secondary effectiveness outcomes included: the seven individual processes of care that were included in the without-fail measure, the consolidated measure of quality which describes the proportion of care patients received among the processes for which they were eligible (e.g., for a patient who received two processes of care but who was eligible for four processes of care, their consolidated quality measure would be 50%, whereas their without-fail rate would be 0%), and patient outcomes (i.e., 90-day recurrent stroke and 90-day all-cause mortality).

#### Quantitative analysis plan: effectiveness assessment

Generalized mixed-effects models at the patient level with random effects for sites were used to analyze the PREVENT intervention effects on the without-fail rate during the active implementation period compared with the baseline period [40]. For the primary effectiveness analysis, the main comparison was the mean facility without-fail rate across the six sites during the baseline data period versus the active implementation data period; adjusting for wave and site variations. The primary analysis included the first TIA event per patient. In sensitivity analyses, we included all TIA events and we will also excluded patients ≥90-years old (because care for such patients may appropriately not include all of the processes of care which were included in the without-fail rate).

Several secondary effectiveness analyses were pre-specified, including [1]: an examination of how the without-fail rate changed in the PREVENT sites compared with VA facilities matched on the basis of TIA patient volume, facility complexity (i.e., teaching status, intensive care unit level),and baseline without-fail rate (with six controls for each intervention site); this analysis allowed for consideration of temporal changes in care [2]; an examination of individual processes of care across the six sites from the baseline period to active implementation period (e.g., how did receipt of high or moderate potency statins change from baseline to active implementation) [3]; an assessment of change in the consolidated measure of quality from baseline to active implementation; and [4] a comparison of the 90-day recurrent stroke rate and the 90-day all-cause mortality rate, before versus after active implementation. For each of these secondary analyses, the multivariable models included adjustment for wave site variations, and baseline comorbidities. Specifically, individual risk-adjustment models were created for each process of care and for each patient outcome. The individual processes of care, the consolidated measure of quality, the 90-day recurrent stroke rate, and the 90-day mortality rate were considered secondary outcomes because the stepped-wedge study was designed to have adequate power (see Sample Size section below) to identify differences in the primary effectiveness outcome (the without-fail rate) and not the secondary outcomes.

#### Mixed methods evaluation plan: user satisfaction, implementation and sustainability assessments

Table 1 summarizes the qualitative data collection methodology including: semi-structured interviews, observations, and Fast Analysis and Synthesis Template (FAST) facilitation tracking [41]. Interviews were conducted in-person during site visits or by telephone at baseline, 6-months and 1-year after active implementation, and at the end of sustainability. Key stakeholders included staff involved in the delivery of TIA care, their managers, and facility leadership; we also accepted “snowball” referrals from key stakeholders. Upon receipt of verbal consent, interviews were audio-recorded. The audio-recordings were transcribed verbatim. Transcripts were de-identified and imported into Nvivo12 for data coding and analysis. Using a common codebook, two team members independently coded identical transcripts for the presence or absence of CFIR constructs as well as magnitude and valence for four selected CFIR implementation constructs (i.e., Goals & Feedback, Planning, Reflecting & Evaluating, and Champions). The project team met to review and discuss similarities and differences in the coding until a shared understanding of each item in the codebook was developed. In addition to the interview data, the study team conducted formal debriefings after each kickoff, site visit, and collaborative call. These observations were recorded and transcribed for analyses. We also used the FAST template, which is a structured electronic log, as a rapid, systematic method for extracting key concepts across data sources including interviews, collaborative calls, and Hub utilization data [41]. We adapted an external facilitator tracking sheet to prospectively collect the dose and contents of external facilitation provided by the study team to participating facility teams [42]. We evaluated local organizational culture using the Organizational Culture Assessment Instrument [43, 44]. Finally, we collected audience-response system (ARS) feedback and written evaluations about program components during kickoffs.

**Table 1:** Qualitative Data Collection Plan

FOCUS OF INQUIRY	DATA	PERIOD	METHOD	PARTICIPANTS
Structure: TIA protocol; TIA providers Process: how clinical teams use data to improve quality; local context	Formal, semi-structured, qualitative interviews	Baseline 6 months into active implementation 12 months into active implementation Sustainability	Audio-recorded & transcribed interviews	Providers who care for and support patients with TIA
Structure: Team composition Process: Team formation; impact evaluation; action planning	Observations of team kickoffs for active implementation	After baseline at the start of active implementation		
Structure: Clinical providers’ attendance and participation Process: Community of care interactions; implementation progress	Observations of Virtual Collaborative Calls	Monthly 1 Hour Calls		
Structure: Local front-line providers involved in TIA care Process: Team dynamics; implementation progress; use of data	Observations of facility visits	Post Visit Debriefings	Audio-recorded & transcribed interviews & field notes	
Structure: Role and service of key informants Process: Use of implementation strategy; implementation progress	FAST* template: a rapid, systematic method for capturing key concepts across data sources	Project Duration	FAST Template	
Structure: Facility team members engaged in quality improvement Process: Facilitation contents and dose	External Facilitation Tracking Sheet		FAST Template and Facilitator notes	Providers who locally adapt PREVENT to improve quality of TIA care

*FAST refers to the Fast Analysis and Synthesis Template [41]

#### Users’ assessment of the program

The assessment of satisfaction with the PREVENT program was evaluated using interview data, ARS, and survey data. Satisfaction was defined as program acceptability, the perception among front-line implementers that PREVENT was palatable or satisfactory based on content, complexity, or comfort. We derived the users’ assessment of the intervention using the intervention characteristics domain from CFIR. We sought to identify the components of the intervention that were most useful or most important to the facility team members.

#### Implementation outcomes and evaluation

PREVENT employed three primary implementation strategies [1]: team activation via audit and feedback, reflecting and evaluating, planning, and goal setting [2]; external facilitation; and [3] building a community of practice. In addition, PREVENT allowed for local adaptation of the intervention components and took advantage of peer pressure while providing facilitation support to the site champion. The two primary implementation outcomes were the number of implementation activities completed during the one-year active implementation period and the final level of team organization (defined as the Group Organization [GO Score]) [45, 46] for improving TIA care at the end of the 12-month active implementation period. The number of implementation activities completed was scored for each site by the research team using a rubric designed for PREVENT. The GO Score [45, 46] was a measure of team activation on a 1–10 scale for improving TIA care based on specified provider practices. Scores between 1 and 3 denoted a beginning level of organization with no facility wide approach, 4–5 reflected a developing approach, 6–7 denoted basic proficiency, 8 indicated intermediate proficiency, and 9–10 reflected a TIA system that was implemented facility-wide and that could sustain key personnel turnover**.**

Using a mixed-methods approach grounded in the CFIR, we examined and evaluated the degree to which the sites engaged in the three primary implementation strategies; the association between implementation strategies and implementation success; contextual factors associated with implementation success; the association between implementation strategies and the without-fail rate; and the association between implementation outcomes and the without-fail rate. In addition, we described the dose, type, and temporal trends in external facilitation that was provided to each site during active implementation.

#### Sustainability evaluation

The sustainability analysis included both a comparison of the change in the without-fail rate from the baseline data period to the sustainability period and from the active implementation period to the sustainability period. We constructed mixed-effects models accounting for random effects for sites as described above for the effectiveness evaluation and explored whether sites with the greatest use of their own quality data demonstrated the greatest program sustainability.

### Site selection

Sites were invited to participate on the basis of demonstrated gaps in quality of care; specifically, if they had baseline without-fail rates of < 50%. All VA acute care facilities with at least ten eligible TIA patients per year were rank ordered in terms of the without-fail rate. Invitations were sent via email beginning with facilities with the greatest opportunity for improvement. Recruitment continued until six facilities agreed to participate. Although some stepped-wedge trials randomly assign facilities to waves (for example in a cluster randomized controlled trial design), PREVENT sites were allocated to waves pragmatically based on the ability to schedule baseline and kickoff meetings.

### Power & Sample Size

**Fig. 4:**
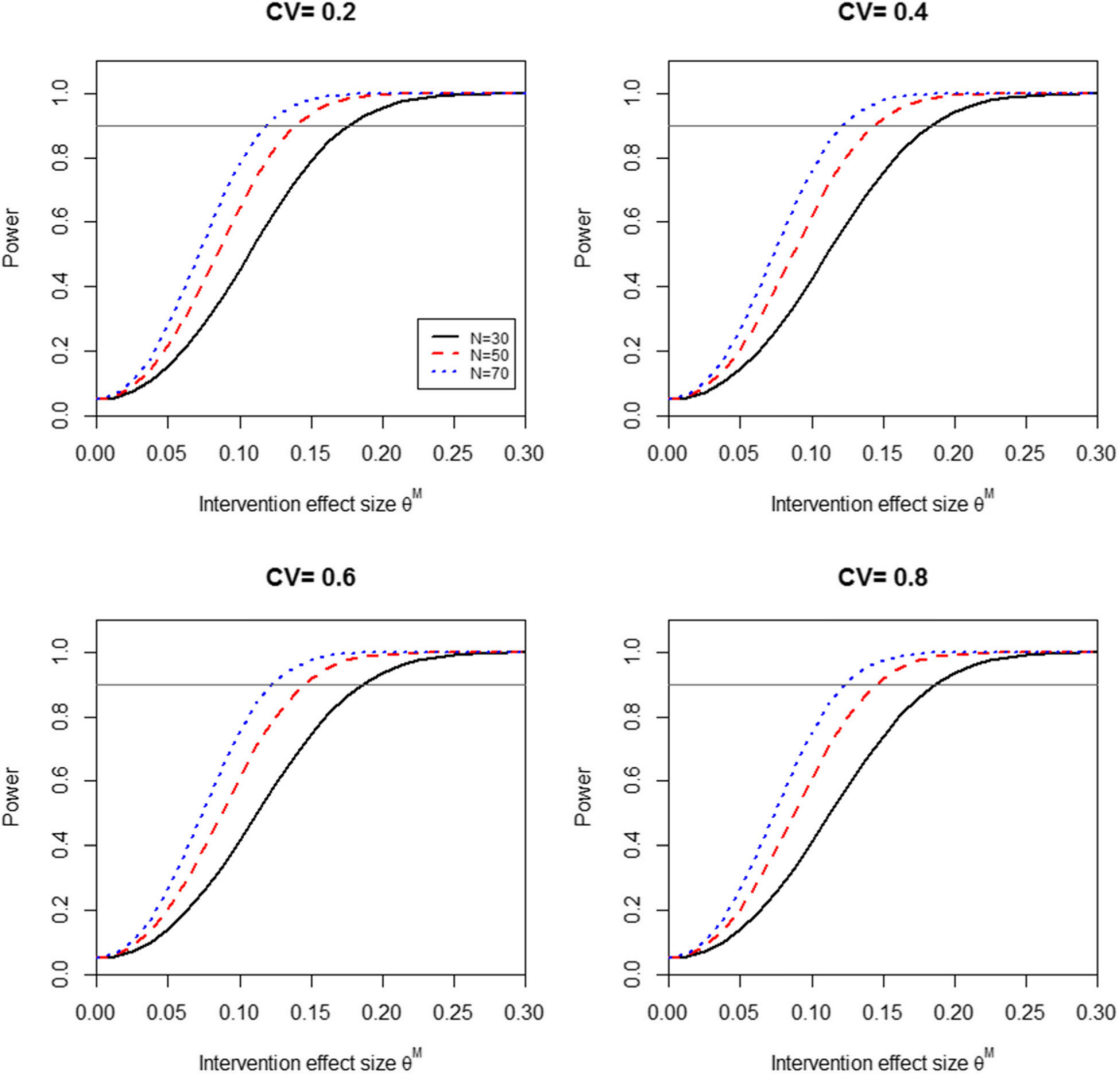
PREVENT Sample Size Design. The panels display how the power curves vary with changes in the coefficient of variation (CV)

The methods used for the sample size design and power calculation for this stepped-wedge trial have been reported elsewhere [36]. Briefly, the six-site, stepped-wedge design provided > 90% power to detect an improvement in the mean facility without-fail rate from 25% during the baseline period to > 45% during the active implementation period. The goal for the sample size was to recruit sites with ≥50 TIA patients per year; however, power was preserved with ≥30 TIA patients per year. Fig. 4 provide the plots of the power for testing the intervention effect *H*_0_ : *θ* = 0, *H*_1_ : *θ* = *θ*^*M*^ for the intervention effect size *θ*^*M*^ ranging from 0.1 to 0.3; allowing for a potential decrement in intervention effect over time. Specifically, we hypothesized that the effectiveness of the intervention would be more robust during the active implementation phase and less robust during sustainability. The panels in Fig. 4 were based on a total of 6 sites (where a site was a single VA facility) with a site size of 30, 50 and 70 (TIA patients cared for at a single VA facility). The results demonstrated reasonable power (greater than or equal to 0.90) for detecting the intervention effect when the effect size is at least 0.20 with a site size no less than 30. The coefficient of variation (CV) was set at 0.2, 0.4, 0.6, and 0.8 to cover a wide range of the between-site variation. The CV seemed to have little effect on power (Fig. 4).

## Results

We have described the development, implementation, and evaluation of a multi-component provider-facing QI intervention to improve TIA care at six VA facilities [22–24]. Results of the planned analyses will be submitted for peer-review as those data become available.

## Discussion

Key strengths of the approach to developing this QI program involved grounding the program in data from multiple sources including interview data to understand the needs of front-line providers across a diverse set of facilities and across disciplines [15]; validation evidence identifying processes of care that could be obtained as electronic quality measures which facilitates ongoing performance measurement and scalability [14]; benchmarking data identifying the gaps in care that should serve as targets for quality improvement, especially processes with large opportunities for improvement for large numbers of potentially eligible patients [14]; and evidence from the existing literature about processes of care that are most robustly associated with improved patient outcomes [25–28]. The strengths of the evaluation plan included both the grounding in the CFIR model and the explicit evaluation of implementation strategies across diverse local contexts.

The PREVENT program was positively aligned with the model of the Learning Healthcare System. In the Institute of Medicine’s book *Best Care at Lower Cost*, the Learning Healthcare System was described as an approach where “clinical informatics, incentives, and culture are aligned to promote continuous improvement and innovation, with best practices seamlessly embedded in the delivery process and new knowledge captured as an integral by-product of the delivery experience.” [47] Already recognized as an example of a stand-out organization that harnesses the power of data to improve the health of the populations it serves [48, 49], the VA was the first federal agency to endorse the Learning Healthcare System’s core values. The design of PREVENT advanced three aspects of a Learning Healthcare System. If PREVENT successfully improves TIA care quality, then we will work with our partners in VA central office to disseminate the program to all VA facilities.

### Learning from data

The PREVENT Hub, unlike static performance dashboards, allowed teams to examine and interact with their performance data to explore hypotheses, plan QI activities, and evaluate change over time. Although audit and feedback has been demonstrated to be effective in QI, we have little insight into how teams use data to improve quality [50]. The PREVENT study provided an opportunity to learn how teams use data to inform QI activities. The patient identification tool provided teams with patient-level, actionable information to identify patients in real-time to ensure that every patient received all the care they needed; this tool is generalizable to other time-sensitive clinical conditions where patients seek care in the ED or inpatient settings.

### Learning from each other

Site teams participated in monthly collaborative calls to learn about relevant topics, share strategies for overcoming challenges to providing highest care quality, and cultivate a sense of community. PREVENT teams were multidisciplinary, providing opportunities to learn across disciplines. For example, although the role of pharmacist-delivered care is well recognized for many clinical conditions, it has been underutilized for the care of patients with stroke or TIA. Given that many TIA process of care involve medication management, collaboration with pharmacy staff offers great promise for delivering guideline-concordant care [51].

### Sharing best practices

Facility-based teams shared tools and best practices in a rich and growing library of diverse resources.

Several limitations of the PREVENT program merit description. The primary limitation of PREVENT was the implementation only within VA hospitals which have the benefit of a unified electronic health record. If this program is found to be effective, then future research should evaluate its implementation in non-VA settings. Second, because several implementation strategies were deployed, it may be difficult to disentangle the unique effects of each strategy. However, we designed multiple data collection sources to capture the effects of each implementation strategy on implementation success using rigorous evaluation methodology. Third, making a diagnosis of TIA can be clinically challenging and some patients who receive a diagnosis code for TIA may well have an alternative diagnosis. Although we know that some of the patients who were coded as having a TIA did not have actually had a TIA, we have neither observed differential miss-classification either across facilities nor across time [30]. In other words, potential TIA miscoding is likely to exist across all of the sites and will likely exist during baseline, active implementation, and sustainability phases. Therefore, it is unlikely that differential TIA miscoding will bias the examination of the effect of the intervention. If, however, the TIA miscoding rate was unexpectedly high, and patients were not getting TIA processes of care because they did not actually have a TIA, then the without-fail rate would be appropriately low. In this case, our ability to detect a change in the without-rail rate would be impaired. Fourth, although a six-site sample was sufficient to provide adequate power, future studies might include a larger number of facilities. Fifth, the PREVENT program targeted clinical teams at the participating sites; the clinicians were the subjects of the implementation and satisfaction evaluations. Future studies should consider how best to include patients’ perspectives in implementation evaluations. Finally, although we plan to deploy the program nationwide if the effectiveness analyses indicate that PREVENT improves TIA care quality, an assessment of scalability during national deployment is beyond the scope of the planned PREVENT research activities.

The promise of Learning Healthcare Systems involves the development of QI programs that are data-driven, meet the needs of stakeholders, and dynamically adapt to changes in performance and context. As illustrated by the PREVENT trial, that promise should likewise extend to program development and evaluation to assess not only *whether* a program works but also *how* and *why* it works.

## Data Availability

These data must remain on Department of Veterans Affairs servers; investigators interested in working with these data are encouraged to contact the corresponding author.

## Abbreviations

CFIR: Consolidated Framework for Implementation Research
ED: Emergency Department
FAST: Fast Analysis and Synthesis Template
GO Score: Group Organization Score
PREVENT: Protocol-guided Rapid Evaluation of Veterans Experiencing New Transient Neurological Symptoms
QI: Quality improvement
SQUIRE: Standards for Quality Improvement Reporting Excellence
TIA: Transient ischemic attack
VA: Department of Veterans Affairs
